# IL-1 Family Cytokine Regulation of Vascular Permeability and Angiogenesis

**DOI:** 10.3389/fimmu.2019.01426

**Published:** 2019-06-25

**Authors:** Erin Fahey, Sarah L. Doyle

**Affiliations:** ^1^Department of Clinical Medicine, School of Medicine, Trinity College Dublin, Dublin, Ireland; ^2^Trinity College Institute of Neuroscience, Trinity College Dublin, Dublin, Ireland; ^3^Our Lady's Children's Hospital Crumlin, National Children's Research Centre, Dublin, Ireland

**Keywords:** IL-1, IL-18, IL-33, IL-36, angiogensis, vascular permeability

## Abstract

The IL-1 family of cytokines are well-known for their primary role in initiating inflammatory responses both in response to and acting as danger signals. It has long been established that IL-1 is capable of simultaneously regulating inflammation and angiogenesis, indeed one of IL-1's earliest names was haemopoeitn-1 due to its pro-angiogenic effects. Other IL-1 family cytokines are also known to have roles in mediating angiogenesis, either directly or indirectly via induction of proangiogenic factors such as VEGF. Of note, some of these family members appear to have directly opposing effects in different tissues and pathologies. Here we will review what is known about how the various IL-1 family members regulate vascular permeability and angiogenic function in a range of different tissues, and describe some of the mechanisms employed to achieve these effects.

## The IL-1 Family of Cytokines

**Figure 1:**
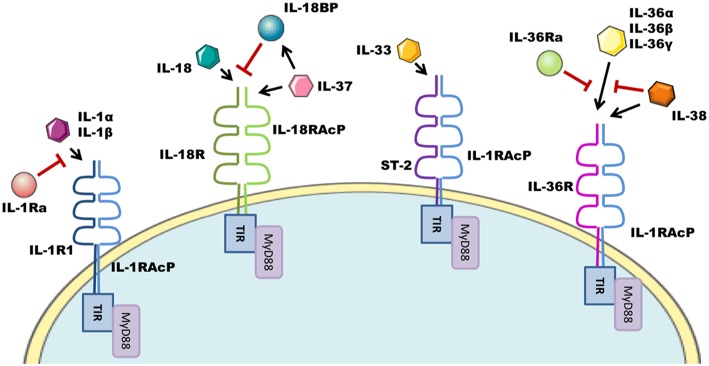
IL-1F family signaling complexes. The IL-1R family has four signaling complexes; IL-1R1/IL-1RAcP, IL-18R/IL-18RAcP, ST-2/IL-1RAcP, and IL-36R/IL-1RAcP. IL-1α/IL-1β signal through IL-1R1/IL-1RAcP; this pathway can be antagonized by IL-1Ra. IL-18 signaling uses the IL-18R/IL-18RAcP complex. IL-18 ligand can be sequestered and thereby inhibited by IL-18BP. IL-37 also utilizes this complex. ST-2/IL-1RAcP is the receptor complex for the IL-33 cytokine. IL-36α/IL-36β/IL-36γ signal through the IL-36R/IL-1RAcP complex, which can be antagonized by IL-36Ra and IL-38.

The interleukin-1 family (IL-1F) are pivotal regulators of the innate immune response composed of 7 agonist signaling ligands [interleukin-1 alpha (IL-1α), interleukin-1 beta (IL-1β), interleukin-18 (IL-18), interleukin-33 (IL-33), interleukin-36 alpha (IL-36α), interleukin-36 beta (IL-36β), and interleukin-36 gamma (IL-36γ)]; three receptor antagonists [interleukin-1 receptor antagonist (IL-1Ra), interleukin-36 receptor antagonist (IL-36Ra), and interleukin-38 (IL-38)] as well as one other cytokine [interleukin-37 (IL-37)] (1, 2) whose biological role remains somewhat controversial. These cytokines signal through the IL-1 receptor (IL-1R) family of transmembrane proteins, whose 11 members form six receptor chains, resulting in four functional signaling complexes (Figure 1). Members of the IL-1R family are grouped due to the characteristic motifs shared in their extra- and intracellular domains. The extracellular domain is typically composed of three immunoglobulin (Ig)-like domains. Interleukin-18 binding protein (IL-18BP) and Toll/IL-1R8 (TIR8) are exceptions having only a single Ig-like domain. Intracellularly, the signaling complexes are identifiable by their (**T**oll/**I**L-1 **R**eceptor) TIR-domains, which are required to recruit the adapter protein myeloid differentiation primary response protein 88 (MyD88) through homotypic interactions and initiate the downstream signaling cascades that are the consequence of IL-1F receptor activation (3). The signaling complexes and their respective ligands can be seen in Figure 1. Most of the IL-1 family cytokines, including IL-1, IL-18, and IL-36 are produced in inactive precursor forms, that require N-terminal cleavage in order to become activated (4, 5). In the case of IL-1 and IL-18, this cleavage is classically performed by caspase-1, that itself is activated by the formation of an inflammasome; however alternative cleavage of pro-IL-1β via various serine proteases and matrix metalloproteinases, and cleavage of IL-18 by caspase-8 has also been reported (5, 6). The mechanisms through which other IL-1 family cytokines, such as IL-33 and IL-36, are cleaved, are still emerging, with studies thus far suggesting that neutrophil elastase and various cathepsins are the relevant proteases (7, 8). Broadly generalizing, upon IL-1 family cytokine binding to its receptor complex, the adaptor protein MyD88 is recruited to the intracellular TIR domain which forms a complex with interleukin-receptor associated kinase 4 (IRAK4), facilitating the activation of the downstream signaling cascade, canonically comprising of mitogen associated protein kinase (MAPK) and Inhibitor of κB Kinase (IKK) complexes, and resulting in the respective activation of Activator protein-1 (AP-1) and nuclear factor kappa-light-chain-enhancer of B cells (NFκB) transcription factors, driving gene transcription of IL-1F responsive genes.

## Angiogenesis and Vascular Permeability

Angiogenesis is the growth of new capillaries from existing blood vessels; a process controlled by complex interactions of inhibitors and activators, an appropriate balance of which is required to initiate and maintain physiological homeostasis. This process is important in development, as well as for tissue growth and repair. However, aberrant angiogenesis or neovascularisation has been linked to several malignancies, including cancer, rheumatoid arthritis (RA), blindness, and psoriasis (9). In many of these conditions, unwanted, disease-associated neovascularisation results in malformed, immature, and unstable vessels that leak plasma factors and can create an oedemic environment. A thorough understanding of the factors that influence the formation of the vasculature in addition to the maturation and integrity of the neovesssels is essential for delineating therapeutic targets for these conditions (10, 11). The process of angiogenesis is multistep, and involves the degradation of the extracellular matrix (ECM), migration, differentiation, and proliferation of endothelial cells (ECs), microtubule formation, and the sprouting of new capillary branches (12). Factors controlling angiogenesis may be categorized as indirect, in that they act via intermediary mechanisms, or direct, in that they are able to induce proliferation, migration and/or differentiation of endothelial cells directly (13).

Current therapeutic options for neovascular and oedemic disease attempt to target the pathways that coordinate angiogenesis and permeability. The best characterized of these is the vascular endothelial growth factor (VEGF) pathway. VEGF is considered the master regulator of angiogenesis and permeability, and is implicated as a driver of neovascularization and oedema in a number of diseases, hence anti-VEGF therapeutics are currently utilized in diseases ranging from age-related macular degeneration (AMD) to a number of forms of cancer (14, 15).

It has long been established that IL-1 is capable of simultaneously regulating inflammation and angiogenesis, indeed one of IL-1's earliest names was haemopoeitn-1 due to its pro-angiogenic effects (16). In fact, proteome profiling has revealed that there are considerable overlaps in pathways and biological functions regulated by IL-1β and VEGF in activated EC, in particular the mitogen activated protein kinase (MAPK) cascade is induced by both IL-1β and VEGF, and may potentially play a role in the overlapping effects caused by inflammatory and angiogenic signaling (17). Other IL-1 family cytokines are also known to have roles in mediating angiogenesis, either directly or indirectly. Of note, two of these family members appear to have directly opposing effects in different tissues and pathologies. Here we will review what is known about how the various IL-1 family members regulate vascular permeability and angiogenic function in a range of different tissues, and describe some of the mechanisms employed to achieve these effects.

## IL-1—An Extensive Mediator of Angiogenesis

IL-1α and IL-1β ligands have been recognized for some time as cytokines that can drive an angiogenic phenotype *in vivo*. IL-1α is rarely secreted by living cells, and has been less widely studied than its homolog, IL-1β. However, there is still a wealth of literature that suggests IL-1α can play a role in mediating angiogenesis, most predominantly in cancer and in the brain (18).

One area in which IL-1α and IL-1β are thought to act synergistically is in the process of wound healing. IL-1α and IL-1β are markedly induced 12–24 h post-wound, and the levels of these cytokines return to basal levels once the proliferative stage of wound healing has been completed (19). The wound healing response involves a finely tuned, self-limiting series of cellular and molecular events orchestrated by the transient activation of specific signaling pathways. Controlled regulation of these events is essential. Failure to initiate key steps at the right time delays healing, leading to chronic wounds, while aberrant initiation of wound healing processes may produce cell behaviors that promote cancer progression. Angiogenesis is a crucial step in wound healing, as there is a substantial oxygen-demand in this highly energy-consuming process (20).

In response to a wound in the brain, a rapid induction of inflammatory cells and astrocytes occurs, IL-1 is among the inflammatory cytokines produced in this process, and it is known to stimulate astrocytosis and neovascularization (21). IL-1α is widely acknowledged to have a role as a direct regulator of angiogenesis following ischemic injury (22). Post-stroke, controlled angiogenesis is important for repairing the damaged area of the brain and is known to improve functional outcome. In post-stroke mice, IL-1α is upregulated, and IL-1α can potently induce the expression of other pro-angiogenic cytokines such as chemokine (C-X-C motif) ligand 1 (CXCL1) in brain EC's, as well as being capable of directly driving proliferation, migration and tube formation, all hallmarks of angiogenesis, in these same cells (23, 24). Such is the potency of IL-1α in this context, downstream targets of IL-1α are also being studied as potential neurotherapeutic molecules in stroke. Perlecan is one such downstream target (25, 26). When Perlecan domain V was systemically administered for 24 h post stroke in rat and mouse models, it increased VEGF levels via α5β1 integrin, and displayed neuroprotective and angiogenic effects (27).

Like IL-1α, IL-1β can mediate angiogenesis in the cerebrovascular system. At least one mechanism likely involves IL-1β upregulation of pentraxin 3 (PTX3), a marker of stroke (28). PTX3 KO mice display reduced post-stroke angiogenesis, and recombinant PTX3 can induce hallmarks of angiogenesis including cell proliferation and tube formation in the murine brain derived endothelial cells; indicating that this IL-1β regulated molecule can mediate angiogenesis and contribute to recovery after stroke (29). However, IL-1 activation in the brain is not without its downsides, and has been shown to be a major driver of neuroinflammation; responsible for activating endogenous microglia and vascular EC, allowing them to recruit peripheral leukocytes that can sustain neuroinflammation (30). In particular, IL-1β can influence neutrophil-mediated toxicity, and neutrophil recruitment to the brain appears to be IL-1β dependent. Transendothelial migration of neutrophils across IL-1β stimulated brain endothelium pushes neutrophils to acquire a neurotoxic phenotype (31).

Perhaps not surprisingly, one of the ways in which IL-1R signaling mediates angiogenesis is indirectly, via its ability to induce VEGF expression and secretion in a number of cell types (32). Under normoxia, IL-1β has been shown to induce hypoxia-inducible factor-1α (HIF-1α) at protein level, despite no change being detected in HIF-1α mRNA levels (33). Given the recent literature on inflammation induced regulation of metabolic processes, this is likely due to IL-1 induced stabilization of the labile HIF-1α protein in response to a metabolic shift in the cell. HIF-1α mediates angiogenesis via its target gene, VEGF, which has established hypoxia regulatory elements in its promoter (34, 35). In fact, early reports demonstrated that IL-1β can drive transcription of both VEGF and its receptor VEGF-receptor 2 (VEGFR2) in cardiac myocytes and cardiac microvascular endothelial cells (CMVEC) (36) indicating that an important role for IL-1 signaling is likely that of potentiating VEGF biology. Later reports demonstrated that IL-1β KO mice have impaired VEGF-controlled mobilization of endothelial progenitor cells into the peripheral blood resulting in markedly reduced neovascularisation compared to wild type mice following ischemic injury (37). There have since been several studies that investigate which pathways are important for IL-1β mediated VEGF induction. In myocardial neovascularisation, the VEGF-dependent physiological response to myocardial ischemia, it has been demonstrated that in addition to HIF1- α stabilization, VEGF mRNA transcription, and stability are improved following incubation with IL-1β; and that this activity is dependent on specificity protein 1 (SP1) sites in the VEGF gene's promoter targeted by IL-1β-activated p38 MAPK and c-JUN N-terminal kinase (JNK) signaling (38). Similarly, treatment with IL-1β of vascular smooth muscle cells (VSMC) from small tumor vessels, also resulted in improved VEGF transcription and stability in a p38 MAPK dependent manner (39). Taken together, these findings indicate that both IL-1β induction of the p38 MAPK signaling cascade and induction of HIF1- α are important for upregulating VEGF, and that IL-1β upregulation of VEGF consequently drives neovascularisation.

It is often reported that cancers are “wounds that never heal” or even “over-healing wounds” (40, 41) and inflammation, the arm of the immune system that regulates wound healing, can be a cause of cancer. In addition to the modulating effect that IL-1 has on VEGF expression and wound healing, IL-1 has also been reported to directly regulate tumor-mediated angiogenesis (18), potentially providing an alternative or adjunct therapeutic target in these cancers. Observationally, studies have demonstrated that IL-1α is secreted by colonic, gastric, and pancreatic cancer cells and that this enhances angiogenesis *in vitro* (42–44). Others have delved further into the mechanism investigating how IL-1α is activated in cancer cells and how this effects neovascularisation and subsequent tumor growth. In gastric cancer cells, N-myc downstream regulated gene 1 (NDRG1) increased IL-1α expression, which was then capable of driving tumor angiogenesis via a c-JUN N-terminal kinase (JNK)/AP-1 dependent pathway (45). In prostate cancer, Bone morphogenic protein-6 (BMP-6) is overexpressed, and was shown to induce IL-1α in macrophages via NFκB/Smad1 signaling (46) that was capable of driving tube formation in endothelial progenitor cells. Tumor growth and neovascularisation is significantly decreased when BMP-6 is expressed in IL-1α knockout (KO) mice compared to wild type controls; indicating that IL-1α is mediating angiogenesis in this context. While it remains possible, and probable, in these latter studies that IL-1 is not working alone, but is also driving VEGF production and effecting neovascularisation in an indirect manner, it is clear that IL-1 is an inducer of angiogenesis in the tumor environment. In melanoma, IL-1α and IL-1β are both required for NFκB activation that results in the upregulation of proinflammatory cytokines IL-6 and IL-8, as well as the adhesion molecules intercellular adhesion molecule-1 (ICAM-1), vascular cell adhesion molecule-1 (VCAM-1), and tissue factor (TF) in EC's, driving these cells toward a proinflammatory phenotype that supports tumor angiogenesis (47). While in Lewis lung carcinoma, IL-1β drives tumor growth through its upregulation of VEGF and the proangiogenic chemokine (C-X-C motif) ligand 2 (CXCL2) (48). Further evidence to support the role IL-1 signaling plays in driving tumor angiogenesis can be seen when the IL-1 signaling pathway is blocked; for example through continuous delivery of IL-1Ra, which neutralizes IL-1 signaling, and has been shown to reduce angiogenesis and tumor development (49). It is also worth noting that initial findings from a major randomized trial [Canakinumab Anti-Inflammatory Thrombosis Outcomes Study (CANTOS)] studying Canakinumab, an IL-1 inhibitory antibody, reported a reduction in the number of incident cases of lung cancer in the treatment groups compared to control groups. Although the mechanisms at play in this setting remain unknown and maybe independent of IL-1 induced neovascularisation. For a deeper review of how IL-1 cytokines regulate cancer we would direct the reader to the review on IL-1 family and Cancer also in this special topic.

A common theme when examining the regulation of angiogenesis by IL-1 family members is the interplay between IL-1 family regulation of and by macrophages, and how this interplay can control angiogenic processes. For example, in Lewis lung carcinoma in addition to IL-1 receptor induction of proangiogenic chemokine CXCL2 driving tumor promoting angiogenesis, IL-1β driven neovascularization was found to be dependent on infiltrating cyclooxgenase 2 (COX-2) positive macrophages (50). Macrophages play a pivotal role in restoring tissue homeostasis after inflammation or damage, and angiogenesis is an integral process in tissue repair, so it is perhaps unsurprising to see that IL-1 family members derived from macrophages have an intrinsic ability to regulate angiogenesis. Matrigel plugs, containing the supernatants from activated macrophages, have been used to demonstrate that macrophage derived IL-1, predominantly IL-1β, attracts myeloid cells from the bone marrow, and these cells locally produced additional IL-1, which further activated macrophages and stimulated EC's to produce VEGF (51). In macrophages at least, it appears that the activation of NFκB, a target of IL-1 signaling, is crucial for VEGF production (52). In a dysregulated or diseased setting, Foucher et al. proposed a mechanism, in which IL-1α, constitutively expressed by interleukin-34 (IL-34) induced macrophages, allows these macrophages to maintain local inflammation, which contributes to aberrant angiogenesis (53).

Angiogenesis is a pathological feature of RA (54). RA synovial tissue, which is rich in blood vessels, invades the periarticular cartilage and bone, resulting in the destruction of the joint. In contrast to IL-1's direct role in mediating angiogenesis in the brain following stroke. IL-1β has no direct effect on mediating angiogenesis in RA, instead it indirectly mediates the pathophysiological angiogenesis via several of its downstream effectors upregulating the proangiogenic mediators angiopoietin-1 (ang-1), Tie-2, and VEGF in a JNK and p38 MAPK dependent pathway (55). IL-1β also upregulates expression of VEGF and chemokine (C-C motif) ligand 21 (CCL21) in RA synovial fibroblasts, this chemokine binds to its receptor C-C chemokine receptor type 7 (CCR7) on EC's, facilitating cell migration, capillary tube formation and *in vivo* blood vessel formation (56, 57). Consequently in the clinic, anakinra, the IL-1Ra homolog treatment that blocks IL-1 signaling, has been shown to reduce neovascularisation of the pannus in RA (58, 59).

The majority of studies described have focused on the effects of IL-1 on VEGF, as the master regulator of angiogenesis and permeability, however, there is also some evidence that suggests IL-1β can regulate other pro-angiogenic growth factors in addition to VEGF. For example, stimulation of corneal endothelial cells (CEC) with IL-1β resulted in the NFκB-dependent induction of fibroblast growth factor-2 (FGF-2), which stimulates endothelial mesenchymal transformation (EndoMT) (60). EndoMT involves EC's losing their endothelial phenotype and attaining a myofibroblast or mesenchymal phenotype (61). While this is not directly related to angiogenesis, it is a cause of many fibrotic conditions and demonstrates the powerful effects IL-1β can exert on endothelial cell regulation. Table 1 details clinical trials targeting IL-1 family cytokines in various diseases that have vascular dysfunction as an aspect of the disorder. It is noteworthy that all but one of these trials are targeting IL-1 with the intention of inhibiting its activity.

**Table 1:** Table of clinical trials targeting IL-1F cytokines in diseases related to vascular dysfunction.

**Condition**	**Treatment**	**Clinical trial.gov identifier**	**Status**
Rheumatoid arthritis	Anakinra	NCT00037700	Phase II, completed—no results available
		NCT00117091	Phase III, completed—no results available
	ACZ855/Canakinumab	NCT00619905	Phase I/II, completed—no results available
		NCT00505089	Phase I/II, terminated—results available
		NCT00504595	Phase II, completed—results available
		NCT00487825	Phase II, completed—results available
		NCT00424346	Phase II, completed—results available
	GSK1827771	NCT00539760	Phase I, completed—no results available
Juvenile rheumatoid arthritis	ACZ855/Canakinumab	NCT00426218	Phase I/II, completed—no results available
Systemic juvenile idiopathic arthritis (Still's disease)	Anakinra	NCT00339157	Phase II/III, completed—no results available
		NCT00037648	Phase II, completed—no results available
		NCT03932344	Enrolling by invitation
	ACZ855/Canakinumab	NCT01676948	Phase III, withdrawn
		NCT00889863	Phase III, completed—results available
		NCT00886769	Phase III, terminated—results available
		NCT02296424	Phase III, completed—results submitted
		NCT00891046	Phase III, completed—results available
		NCT02396212	Phase III, completed—results submitted
	Rilonacept	NCT01803321	Phase I, completed—no results available
		NCT00534495	Phase II, completed—results available
Osteoarthritis	sc-rAAV2.5IL-1Ra	NCT02790723	Phase I, recruiting
	Anakinra	NCT00110916	Phase II, completed—no results available
	Diacerein	NCT00685542	Phase IV, completed—no results available
Corneal neovascularization	Topical IL-1Ra	NCT00915590	Phase I/II, terminated (lack of participants)—results available
Wet age-related macular degeneration	ACZ855/Canakinumab	NCT00503022	Phase I, completed—no results posted
Kawasaki disease	Anakinra	NCT02179853	Phase I/II, recruiting
		NCT02390596	Phase II, recruiting
Atherosclerosis/coronary artery disease	Anakinra	NCT01566201	Completed
	ACZ855/Canakinumab	NCT01327846	Phase III, active—not recruiting
	Rilonacept	NCT00417417	Phase II, completed—results available
Giant cell arteritis	Anakinra	NCT02902731	Phase III, not yet recruiting
Chronic renal insufficiency	Rilonacept	NCT01663103	Phase IV, completed—results available
Intracerebral hemorrhage	Anakinra	NCT03737344	Phase II, not yet recruiting
Subarachnoid hemorrhage	Anakinra	NCT03249207	Phase III, recruiting
Abdominal aortic aneurysm	ACZ855/Canakinumab	NCT02007252	Phase II, terminated (lack of efficacy)—results available
Behcet's disease	Anakinra	NCT01441076	Phase I/II, completed—results available
	GSK1070806	NCT03522662	Phase II, not yet recruiting

### IL-18 as an Angiogenic Mediator—the Context is Key

IL-18 signals through its own receptor (IL-18R) and is unique among the other family cytokines in that it has its own specific accessory protein (IL-18RAcP). IL-18 is considered a pro-inflammatory cytokine due to its potency in activating Natural Killer cells and instructing T helper cell differentiation. Like IL-1β, IL-18 requires cleavage from its inactive precursor in order to become activated; this cleavage is classically performed by the inflammasome activated caspase-1 (5, 62). IL-18 has not been studied to the extent that IL-1 has in the context of angiogenic regulation, however, the reports in the literature appear opposing, indicating that IL-18 appears capable of mediating either a pro-angiogenic signal or an anti-angiogenic signal dependent on the tissue site. In fact the first report of IL-18 involvement in vascular regulation came in 1999 and described how IL-18 negatively regulated neovascularisation *in vivo* (63), this was quickly followed by a report in 2001 demonstrating that IL-18 was pro-angiogenic as it could induce endothelial tube formation both *in vivo* and *in vitro* (64).

### IL-18 as a Pro-angiogenic Cytokine

In RA, IL-18 is elevated in sera, synovial tissues and synovial fluid of patients compared to healthy controls, and IL-18 can upregulate expression of RA stimulators including the adhesion molecules ICAM-1 and VCAM-1, chemokines and VEGF *in vitro* (65, 66). These factors encourage the recruitment and activation of leukocytes as well as the formation of new blood vessels. In fact, IL-18 has been shown to stimulate human dermal microvascular endothelial cells (HMVEC-d) migration and tube formation in Matrigel plugs in a Src- and JNK- dependent manner (67). Other mechanisms that drive IL-18 mediated angiogenesis have also been elucidated in RA. Chitinase-3-like protein 1 (YKL-40) is a proinflammatory molecule that is strongly expressed in RA patients (68). YKL-40 has the ability to induce IL-18 production in osteoblasts, which in turn stimulates angiogenesis in endothelial progenitor cells in a process that requires the suppression of miR-590-3p via the focal adhesion kinase (FAK)/PI3K/Akt pathway (69). IL-18 has also been associated with IL-1β in RA patient biopsies, raising the possibility that these molecules are working in tandem to drive the aberrant angiogenesis as well as other features of the disease (70). In the rare autoimmune disorder, inflammatory myopathy, hypoxia induced mitogenic factor (HIMF) was found to increase IL-18 production in myoblasts via a phosphoinositide-dependent kinase-1 (PDK1)/PI3K/Akt signaling cascade (71). This IL-18 was secreted and drove tube formation in endothelial progenitor cells as well as driving angiogenesis in *in vivo* models.

One of the better understood ways in which IL-18 exerts angiogenic function is via its effect on macrophages. IL-18 has been shown to act synergistically with IL-10 to amplify the production of osteopontin (OPN) and thrombin, angiogenic mediators in their own right, from macrophages, a process which alters the polarization of M2 macrophages, as characterized by the increased expression of CD163 (72). CD163 could potentially be responsible for mediating cell-cell interactions between these macrophages and EC, thereby resulting in excessive angiogenesis. On a side note, in addition to mediating angiogenesis, macrophage derived IL-18 has also been reported to drive vascular remodeling in inflammageing, a process prevalent in many age-related disorders (73).

### IL-18 as a Negative Regulator of Angiogenesis

Despite the variety of tissues in which IL-18 demonstrates a pro-angiogenic profile, there is clear evidence that indicates this is not the end of the story. In the eye especially, there is a significant body of work that attests to IL-18 acting as an anti-angiogenic factor in different models of disease. IL-18 was initially described as a suppressor of angiogenesis by Cao et al. who reported that IL-18 inhibited the proliferation of bovine CEC's, and suppressed mouse corneal neovascularisation (63). More recently it has been demonstrated in a clinical cohort that individuals with macular oedema due to retinal vein occlusion who have high aqueous levels of IL-18 at baseline have better visual outcomes following anti-VEGF administration than those with low levels of IL-18 at baseline (74). This may be due to IL-18's reciprocal negative regulation of VEGF that is reported in the eye (74, 75). Our own group has demonstrated that IL-18 inhibits choroidal neovascularisation (CNV) in both murine and non-human primate laser induced models of neovascular AMD. Administration of IL-18 to experimentally induced CNV reduced the volume of the CNV lesion significantly compared to vehicle control in both murine and non-human primate models, again adding to the growing data that, in the eye at least, IL-18 has anti-angiogenic properties (75, 76). This finding is further strengthened by experiments that demonstrated in the JR5558 mouse model, which spontaneous develops bilateral CNV and retinal angiomatus proliferation, IL-18 administered via interperitoneal injection suppressed both progression and enhanced regression of the spontaneous CNV (75). Similarly, in VEGF^hyper^ mice, that spontaneously develop CNV lesions physiologically similar to those observed in AMD, the ablation of IL-18 expression resulted in a significant increase in the volume and number of lesions formed, when compared to the VEGF^hyper^ phenotype alone or in combination with inhibition of IL-1β or NLRP3 (77).

In line with data emerging from studies on the eye, IL-18 has also been shown to inhibit neovascularisation in murine fibrosarcoma and suppress growth, an anti-angiogenic function that suggests it may have a role in some contexts as a tumor suppressor (63). IL-18 also suppressed tumor growth and metastasis in implanted Lewis lung cancer, an effect it achieved by downregulating VEGF, thereby suppressing angiogenesis (78). This data implies that IL-18 down regulation of VEGF is not unique to the eye but is observed in non-ocular tissue as well.

Even in the case of macrophage derived IL-18, the pro-angiogenic effects of which have been referred to above; there are instances where IL-18 has been shown to negatively regulate angiogenesis. IL-18 produced by the tumor induced M1 macrophages in spontaneous fibrosarcoma (NFSA) tumors, cause the destruction of EC's *in vitro*, and are suspected to result in the necrosis of NFSA tumors in part by enhancing macrophage phagocytosis (79). This conclusion is supported by research that shows the macrophage derived IL-18 strongly inhibited blood vessel formation in the tumors *in vivo* (80). However, there is abundant evidence to suggest that in many cancers, IL-18 is more harmful than helpful in regard to tumor angiogenesis and metastasis (81). In human gastric cancer cell lines, IL-18 increased cell migration directly, as well as enhancing VEGF induced migration (82). This study also demonstrated positive feedback between IL-18 and VEGF, with VEGF enhancing IL-18 production in a manner dependent on endoplasmic-reticulum kinase-1/2 (ERK 1/2) phosphorylation.

The diametrically opposing reports of IL-18 effector function in the context of angiogenesis are intriguing and it is not clear why or how this can be the case. The overwhelming evidence that IL-1 is pro-angiogenic and the fact that due to their shared processing via caspase-1 cleavage, IL-18 and IL-1 tend to be found together may provide some context to speculate for instance that the balance of molar concentrations of IL-18 and IL-1 *in vivo* may influence the environment such that the ability of IL-18 comes down in favor of an anti-angiogenic signal vs. a pro-angiogenic signal. Alternatively, the timing of IL-18 signaling may be critical, or there may be a threshold of relative abundance of pro-angiogenic signals that IL-18 cannot overcome. These remain conjecture; however, one thing that is clear is that further research into the mechanisms of action of IL-18 in mediating angiogenesis and the relative contributions of macrophage vs. endothelial cell signals are needed in order to help understand how this cytokine can promote both pro-and anti-angiogenic signals.

## IL-33 in the Context of Neovascular Regulation

IL-33 is an IL-1 family member, widely expressed in a number of different tissues, that signals via the ST-2/IL-33 receptor and IL-1RAcP complex and induces T-helper type-2 (Th2) associated cytokines (83, 84). IL-33 is expressed in a wide variety of tissues, including stomach, lung, central nervous system (CNS) and skin, however in these tissues it appears to be confined to endothelial, smooth muscle and epithelial cells (83). The method by which IL-33 is activated is a matter of some debate; originally, like IL-1 and IL-18, it was thought to be cleaved by caspase-1 for activation, however others have suggested that secreted full length IL-33 is itself active and that the caspase-1 cleavage results in the inactivation of the cytokine (85). IL-33 can however, be processed by various serine proteases, into a more biologically active mature form (86, 87). It has also been reported that IL-33 can behave as a nuclear factor; it is the same molecule that was originally called nuclear factor high endothelial venules (NF-HEV), and *in vivo* it associates with heterochromatin and has potent transcriptional repressor properties (88). In EC's, nuclear IL-33 is a marker of quiescence, which is lost in a Notch dependent manner during angiogenesis (89).

On a mechanistic level, similar to the other IL-1 family members, it has not been clearly delineated how soluble IL-33 exerts its effects on angiogenesis. The majority of reports indicate that IL-33 promotes a pro-angiogenic phenotype, however others report that IL-33 signaling has an anti-angiogenic function. Urokinase-type plasminogen activator (u-PA) plays a pivotal role in extracellular proteolysis, and is thought to be involved in the modulation of angiogenesis. IL-33 can upregulate u-PA at both mRNA and protein level in human coronary artery cells and human umbilical vain endothelial cells (HUVEC) in a time- and concentration-dependant manner (90). However, it has yet to be explored whether blocking this function of IL-33 alters its ability to drive angiogenesis in EC.

As a Th2 promoting cytokine, IL-33 has been implicated in asthma pathogenesis. Angiogenesis is a feature of airway remodeling in asthma, and it has been demonstrated that IL-33 can increase the expression of blood vessel von Willebrand factor (vWF), as well as angiogenic factors such as angiogenin when administered nasally to a murine asthma surrogate model (91). IL-33 has also been shown to induce microvessel formation by human EC *in vitro* in a concentration dependent manner (91). In diabetic mice, IL-33 was found to improve wound healing and re-epithelization of skin wounds by promoting new ECM deposition and neovascularisation, and was capable of inducing expression of VEGF and vWF (92). In a similar manner, hypoxic human pulmonary artery endothelial cells (HPAEC), increase s IL-33/ST-2 expression and in this cell type IL-33 enhances proliferation, adhesiveness and spontaneous angiogenesis. This process is dependent on HIF-1α, the aforementioned VEGF transcription factor, as when HIF-1α is knocked down, IL-33 does not mediate its pro-angiogenic effects on HPAEC (93). This report implies IL-33 may be mediating angiogenesis by upregulating VEGF expression through HIF-1α. Nitric oxide (NO) production in EC is transiently regulated by VEGF and Ang-1, in turn NO can modulate the angiogenic function of these factors. It has been shown that IL-33 can activate the PI3K/Akt/endothelial nitric oxide synthase (eNOS) signaling cascade in a TRAF-6 dependent manner; however, the same study demonstrated that despite IL-33's pro-angiogenic properties, it did not stimulate the levels of VEGF in the manner of IL-1β (94).

Like IL-1 and IL-18 there are some reports that demonstrate IL-33 acting as a pro-angiogenic mediator in tumors. Tumor derived IL-33 is capable of inducing tumor angiogenesis by activating EC (95). Currently, the pervading opinion is that IL-33, like IL-18, can have both pro- and anti-tumorigenic functions, as reviewed by Fournie et al. (96) and Brint et al. in this special topic.

One context where IL-33 appears to have anti-angiogenic properties, like IL-18, is in the eye. IL-33 and ST-2 are expressed constitutively in human and murine retina and choroid tissues. Like IL-18, IL-33 has been shown to protect against angiogenesis in the eye. IL-33 appears to regulate tissue remodeling inhibiting CNV formation in an ST-2 dependent manner when injected intravitreally into mouse eyes (97). To date, in contrast to IL-1, IL-33 is not a target in the clinic for diseases with dysregulated angiogenesis (clinicaltrials.gov).

## IL-36, IL-37, and IL-38—The Story Emerging so Far

There are three IL-36 isoforms—IL-36α, IL-36β, and IL-36γ, which signal through the IL-36R and IL-1RAcP complex. As with other IL-1 family members, IL-36 cytokines require cleavage for activation, however this is not performed by caspase-1. Instead the IL-36 cytokines are cleaved by the neutrophil proteases cathepsin G and elastase (7), other reports also indicate that cathepsin-S can cleave the IL-36γ isoform (8). To date, IL-36 has been widely studied in psoriasis, where its proinflammatory effects are strongly associated with disease pathology. Psoriasis lesions show a developed vascular network, with wide, leaky capillaries, and it has been demonstrated that IL-36γ, which is commonly found at high levels in these lesions, can activate both HUVEC and HMVEC-d (98, 99). IL-36γ enhanced angiogenesis in these HUVECs in a VEGF-dependent manner, and conditioned media from macrophages incubated with IL-36γ could activate EC and induce ICAM-1 expression (100). These early studies suggest that at least in psoriasis IL-36γ is acting as a pro-angiogenic mediator. IL-36γ is a recognized T-bet target in myeloid cells, and T-bet can instigate decreases in tumor growth by promoting dendritic cell (DC)-mediated ectopic lymphoid organogenesis in a manner that is dependent on IL-36γ (101, 102). There is the mounting evidence that suggests IL-36 cytokines can act as tumor suppressors, although it is yet to be deciphered whether this function has any connection to angiogenic regulation (103, 104).

IL-37 is considered to be an anti-inflammatory cytokine, as it has the capability to suppress MyD88-dependent inflammatory cytokine expression (105–107). Currently, IL-37 is thought to signal through the IL-18R and a yet unidentified accessory protein (108). IL-37 can act as an intracellular cytokine; it can translocate to the nucleus and interact with SMAD family member-3 (SMAD3) to exert anti-inflammatory function (109). IL-37 has the ability to bind TGF-β, which is a pivotal regulator of both developmental and patho-physiological angiogenesis (110). When IL-37 binds to TGF-β, it enhances binding of IL-37 to the activin receptor-like kinase 1 (ALK1) receptor complex and allows IL-37 to signal through ALK1 to activate pro-angiogenic responses (111). IL-37 is expressed and secreted in EC and upregulated under hypoxic conditions, where it enhances cell proliferation, capillary formation, migration, and vessel sprouting from aortic rings with potency comparable to that of VEGF (112). Interestingly, it appears that IL-37's ability to induce the tube formation characteristic of angiogenesis is strongly dose-dependent. Lower doses of 1 and 10 ng/ml could potently drive tube formation in HUVEC, along with other angiogenic characteristics including proliferation and endothelial migration, but little change occurs upon the higher 100 ng/ml dose, which interestingly was reported by another group to suppress tube formation in the same cell type (112, 113).

IL-37, like many of the other IL-1 family members, has been found in higher levels in serum of RA patients vs. healthy control, and the levels are also higher in patients with more advanced disease (114). It has not been decisively established what function this IL-37 plays yet, but it is worth noting that the other IL-1 family cytokines are known to be pro-angiogenic mediators in RA. Due to IL-37's role as an anti-inflammatory cytokine there is speculation that this elevation may be trying to counter the proinflammatory cytokine profile seen in RA.

IL-38 is a relatively novel IL-1 family member, that signals through the IL-36R and has been shown to have anti-inflammatory properties. In a murine model of oxygen induced retinopathy, treatment with IL-38 reduced the size of neovascular regions, indicating the attenuation of angiogenic processes *in vivo* (115). IL-38 was also shown to attenuate proliferation, migration, and tube formation of EC in a dose dependent manner. IL-38 is strongly expressed in synovial tissues from RA patients, and as IL-38 KO mice show greater RA severity in auto-antibody induced RA, it is tempting to suggest that IL-38 acts as an inhibitor of RA pathogenesis (116).

## Regulation of Vascular Permeability by IL-1 Family Cytokines

The vascular endothelium forms a barrier that regulates the flow of molecules, as well as leukocyte, entry into tissues. To regulate vascular permeability is to regulate the cell-cell junctions between EC. This cell-cell junction has several complexes that regulate and contribute to its integrity, including tight junctions and adherens junctions. Molecules like VEGF can regulate vascular permeability by promoting signaling cascades that alter the composition of the junction. However, it is note-worthy that not all mediators of angiogenesis are also able to alter vascular permeability, and indeed there are well-recognized examples of pro-angiogenic mediators acting as anti-permeability factors, thereby helping to improve the vascular integrity, as is the case for Ang-1 (117). It is well-established that IL-1 family cytokines can alter the permeability of vasculature, and, as is the case with angiogenesis, the different IL-1 family cytokines vary in their functionality, with some members displaying tissue dependent effects.

## IL-1 is a Potent Inducer of Vascular Permeability

With respect to IL-1, it appears that despite IL-1α inducing increases in permeability (118, 119), this isoform has slighter effects than IL-1β, at least *in vitro* (120). One of the mechanisms by which IL-1β has been shown to alter the permeability of the vasculature is by regulating the expression of cell-cell junction components. Of note, treatment with IL-1β can result in the loss of β-catenin and VE-cadherin specifically at the endothelial cell border, which results in small holes and gaps forming between cells (121). IL-1β can also induce vascular permeability indirectly through induction of pro-permeability factor R-spondin 3 (RSPO3) (121). Combined treatment of IL-1β and RSPO3 can synergistically increase permeability in human coronary artery endothelial cells (HCAEC), HPAEC, human cerebral microvascular endothelial cells (HCMVEC), human brain microvascular endothelial cells (HBMVEC), and HMVECd (122).

The largest body of evidence characterizing IL-1's role in regulating vascular permeability comes from literature on the blood-brain barrier (BBB). The endothelium and the associated astrocytes control the balance between barrier stability and permeability via production of factors that regulate vessel plasticity (123) and recombinant IL-1β increases the permeability of the BBB in rats when injected intracranially (124). Interestingly, evidence that suggests IL-1β injection into the striatum of juvenile brains results in a neutrophil dependent increase in BBB permeability, but this effect is not recapitulated in mature animals (125). This study demonstrated that in the juvenile animals the blood vessels that recruited neutrophils displayed loss of the tight junction proteins occludin and zonula occludens-1 (ZO-1) and redistribution of vinculin, an adherens junction component involved in the linkage of integrin adhesion molecules to the actin cytoskeleton (125). Electron microscopy further demonstrated that cell-cell adhesions in these cells are morphologically different to control brain endothelial junctions, implying that IL-1β mediated leukocyte recruitment can result in junction disorganization and BBB breakdown. Additionally, IL-1β intracerebral administration in adult rats induces meningitis, however in juvenile rats that are between 2 and 6 weeks old IL-1β has the secondary effect of increasing BBB permeability (126). Chemokines, including cytokine-induced neutrophil chemoattractant (CINC-1), are induced following the intracerebral administration of IL-1β, and this induction produces a far more intense neutrophil response in juvenile rats compared to their adult counterparts (127). The IL-1β mediated breakdown in BBB integrity in juvenile rats could be attenuated by using a CINC-1 neutralizing antibody, indicating that the difference in response between adult and juvenile mammals may be due to differences in sensitivity to IL-1β upregulated CXC chemokines. The finding that these processes are applicable only to juvenile animals is reflective perhaps of the fact that children are more inclined to encounter permanent damage and mortality than adults after trauma or inflammation in the brain.

In encephalitis, parenchymal brain inflammation, the ratio of IL-1Ra to IL-1β in the cerebrospinal fluid can be indicative of patient outcomes, with a higher ratio of IL-1β to IL-1Ra indicative of poor patient outcome, and associated with reduced integrity of the BBB (128). Loss of BBB integrity is also an early significant event in Multiple Sclerosis (MS) and has been proposed through microarray analysis to induce expression of HIF-1α and VEGF-A in astrocytes, as well as potently downregulating an important maturation and stability factor for BBB integrity gravin/src-suppressed C-kinase substrate (SSeCKs) (129). In co-cultures of astrocytes and HBMVEC, endothelin-1 (ET-1) could induce IL-1β in astrocytes, and this IL-1β was demonstrated to be the effector of ET-1 induced BBB permeability (130).

In a manner akin to its effect on the BBB, IL-1β can alter the permeability of the blood-retina-barrier (BRB), by negatively regulating the tight junctions of the retinal vascular endothelium (131). Mice exposed to retinal cryopexy, followed by intraperitoneal administration of [^3^H]mannitol allowed radioactive leakage to be compared between the retina and the lung/kidney (132). This technique demonstrated that IL-1β caused relatively rapid breakdown of the BRB, and that this breakdown in barrier integrity was more prolonged in nature than that induced by VEGF alone. In a transgenic mouse model, where IL-1β was overexpressed in the lens, there was an early peak of VEGF at P5-P7, which was determined to be IL-1β dependent and coincided with the onset of BRB breakdown, which suggests that similar to angiogenesis, IL-1β can exert some of its regulatory effects on vascular permeability by inducing VEGF expression (133).

In addition to its effects in brain and retinal endothelium, IL-1β induces lung vascular permeability in an integrin αvβ6 dependent manner in a lung injury model (134) and contributes to significant increases in the vascular permeability in dengue, the most virulent haemorrhagic disease worldwide (135). Interestingly though, the potent effect of IL-1β on barrier integrity is not ubiquitous to all endothelial cell types. The glomerular capillaries differ from most endothelium types due to their formation of large fenestrated areas that comprise 20–50% of the entire endothelial surface; the result of this is that human renal glomerular endothelial cells (HRGEC) are naturally more permeable than other endothelial cell types. Treatment of HRGEC with IL-1β therefore results in a modest increase in permeability compared to that seen in HUVEC following the same treatment (136). In addition to the wealth of data on IL-1 effects on the BBB and BRB, this may indicate that the primary effect of IL-1 is on regulating the tight junctions and adherens junctions that are not found in fenestrated capillary beds.

## Regulation of Vascular Permeability by Other IL-1 Family Cytokines

As with angiogenesis, the other IL-1 family members have also been shown to have a level of influence over vascular permeability, although for the more novel members of the family—IL-36, IL-37, and IL-38—their effects, if any, remain to be examined. However, there are studies, all be they limited, that demonstrate that IL-18 and IL-33 can regulate vascular permeability effectively.

Like IL-1, IL-33 has also been shown to regulate the permeability of EC, by altering VE-cadherin. IL-33 increased the permeability of HUVEC, as demonstrated in a [^14^C] sucrose permeability assay *in vitro* and the Miles vascular permeability assay *in vivo*. This increase in permeability coincided with a reduction in the localization of VE-cadherin to the cell-cell junction (94). The authors of this study also demonstrated that IL-33 treatment resulted in the phosphorylation of VE-cadherin in a VEGF-independent manner; VE-cadherin phosphorylation is known to correlate with the loosening of adherens junctions, which is associated with transendothelial permeability. Further studies have demonstrated that IL-33 can also decrease expression of tight junction proteins, such as occludin, in order to decrease the barrier integrity of HUVEC (137).

Similar to its effects on neovascularisation, IL-18 can either promote or restrict permeability in a context dependent manner. IL-18 is upregulated in inflamed lungs, and the mature IL-18 cytokine can be found in bronchoalveolar (BAL) fluid. In a rat model of lung inflammation, caused by deposition of IgG immune complexes, intratracheal administration of IL-18 was shown to significantly increase lung vascular permeability, as well as increasing neutrophil and inflammatory cytokine presence in BAL fluids (138). However, in the retina, IL-18 administration reduces vascular leakage in a spontaneous mouse model of bilateral neovascularisation as measured by fluorescein angiography (75) and likewise in the brain it has been demonstrated that IL-18 can protect against BBB disruption. Status epilepticus (SE) is an epileptic condition in which multiple consecutive epileptic fits occur with no recovery of consciousness between them; resulting in an increase in BBB permeability in limited cerebral regions, this increase in permeability is followed by vasogenic oedema. IL-18 is upregulated following SE in local astrocytes, microglia and macrophages, and it has been shown by infusing recombinant IL-18 into the rat piriform cortex that this IL-18 reduces vasogenic oedema formation, potentially through its upregulation of dystrophin and its possible mediation of BBB permeability (139).

## Concluding Remarks & Questions for Future Research

**Figure 2:**
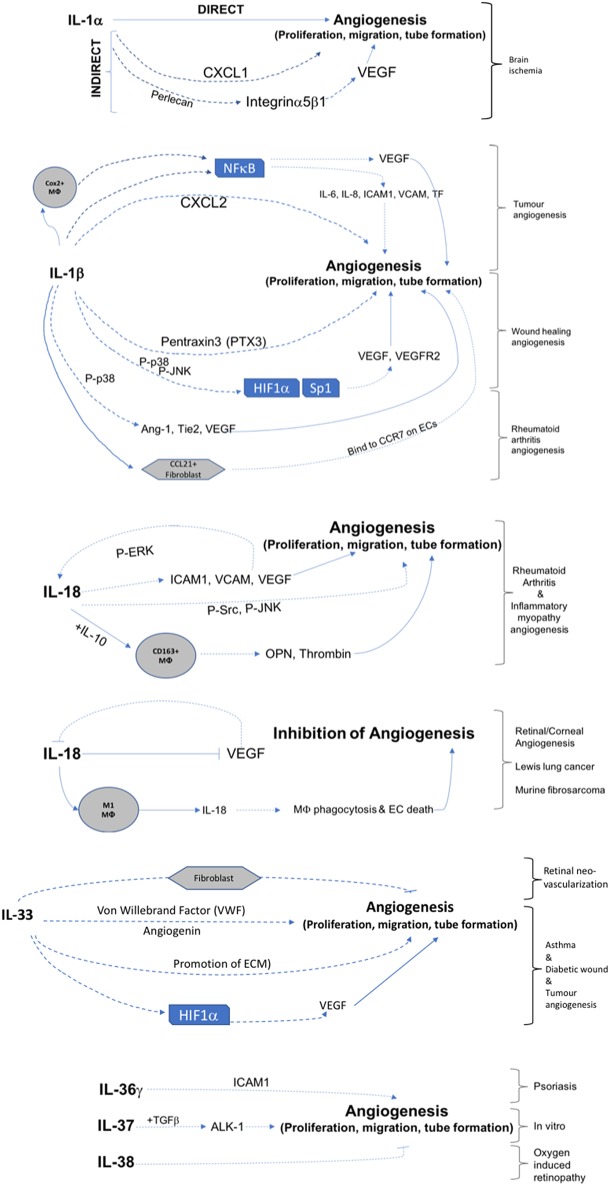
Graphical representation of IL-1F cytokine regulation of angiogenesis. IL-1 F cytokines regulate angiogenesis either by promotion of proliferation, migration, and tube formation or by inhibiting these steps. Direct mechanisms are depicted by solid arrows, indirect mechanisms are depicted by broken arrows. Brackets on the right hand side of the figure indicate mechanisms associated with specific angiogenic diseases. Transcription factors are shown in boxes; *P*, indicates phosphorylation event; Mϕ, macrophage.

**Figure 3:**
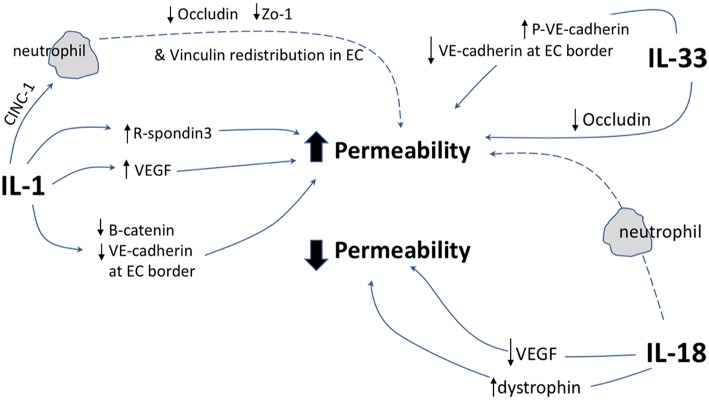
Graphical representation of IL-1F cytokine regulation of vascular permeability. IL-1 F cytokines regulate vascular permeability, direct mechanisms are depicted by solid arrows, indirect mechanisms are depicted by broken arrows. EC, endothelial cell; P, indicates phosphorylation event. ↑ indicates upregulation of protein; ↓ indicates down regulation of protein.

This review has demonstrated that IL-1 cytokine family signaling clearly has an influential role in mediation of angiogenesis and vascular permeability during disease processes, see Figures 2, 3 for graphical representations. For the original members of the family—IL-1α and IL-1β–their proangiogenic effects have been characterized widely and many attempts have been made to understand the drivers and resulting signaling cascades at a molecular level. In the case of IL-18 and IL-33, although their ability to influence angiogenesis is undeniable, questions remain about how they promote angiogenesis in some tissues and yet inhibit it in others. For the more recently discovered members of the family—IL-36, IL-37, and IL-38—studies are beginning to surface that demonstrate that they too, most likely possess the ability to regulate angiogenesis in a number of tissues, yet it remains to be seen as to whether they have the singular effects of IL-1 or the more nuanced effects observed for IL-18 and IL-33. There is equally clear evidence that demonstrates IL-1 can expansively regulate increased permeability of the vasculature throughout the body. IL-18 and IL-33 have also demonstrated that they can alter the cell-cell junction of EC, thereby altering permeability. Whether IL-36, IL-37, and IL-38 can also exert sway over vascular permeability akin to the other IL-1 family members is unknown and remains to be explored. In conclusion, the IL-1 family have potent and far reaching effects on the regulation of angiogenesis and vascular permeability, that are accomplished through both direct and indirect mechanisms.

## Author Contributions

EF wrote the manuscript. SD wrote and edited the manuscript.

### Conflict of Interest Statement

The authors declare that the research was conducted in the absence of any commercial or financial relationships that could be construed as a potential conflict of interest.

## Abbreviations

AP-1: Activator protein-1
ALK1: Activin receptor-like kinase 1
AMD: Age-related macular degeneration
ang-1: Angiopoietin-1
BBB: Blood-brain barrier
BRB: Blood-retina-barrier
BMP-6: Bone morphogenic protein-6
BAL: Bronchoalveolar
CMVEC: Cardiac microvascular endothelial cells
CCR7: C-C chemokine receptor type 7
CNS: Central nervous system
CCL21: Chemokine (C-C motif) ligand 21
CXCL1: Chemokine (C-X-C motif) ligand 1
CXCL2: Chemokine (C-X-C motif) ligand 2
YKL-40: Chitinase-3-like protein 1
CNV: Choroidal neovascularisation
JNK: c-JUN N-terminal kinase
CEC: Corneal endothelial cells
COX-2: Cyclooxygenase-2
CsA: Cyclosporine a
CINC-1: Cytokine-induced neutrophil chemoattractant
DC: Dendritic cell
ERK 1/2: Endoplasmic-reticulum kinase-1/2
EC: Endothelial cells
eNOS: Endothelial nitric oxide synthase
ET-1: Endothelin-1
ECM: Extracellular matrix
FGF-2: Fibroblast growth factor-2
FAK: Focal adhesion kinase
HBMVEC: Human brain microvascular endothelial cells
HCMVEC: Human cereberal microvascular endothelial cells
HCAEC: Human coronary artery endothelial cells
HMVEC-d: Human dermal microvascular endothelial cells
HPAEC: Human pulmonary artery endothelial cells
HRGEC: Human renal glomerular endothelial cells
HUVEC: Human umbilical vain endothelial cells
HIMF: Hypoxia induced mitogenic factor
HIF-1α: Hypoxia-inducible factor-1α
Ig: Immunoglobulin
ICAM-1: Intercellular adhesion molecule-1
IFNγ: Interferon gamma
IL-1: Interleukin-1
IL-1F: Interleukin-1 family
IL-1R: Interleukin-1 receptor
IL-1Ra: Interleukin-1 receptor antagonist
IRAK4: Interleukin-1 receptor associated kinase 4
IL-10: Interleukin-10
IL-18: Interleukin-18
IL-18BP: Interleukin-18 binding protein
IL-33: Interleukin-33
IL-34: Interleukin-34
IL-36: Interleukin-36
IL-36Ra: Interleukin-36 receptor antagonist
IL-38: Interleukin-38
IL-6: Interleukin-6
IL-8: Interleukin-8
JAK2: Janus kinase 2
KO: Knock-out
M-CSF: Macrophage colony stimulating factor
MAPK: Mitogen-activated protein kinase
MAP2K2: Mitogen-activated protein kinase kinase 2
MS: Multiple Sclerosis
MyD88: Myeloid differentiation primary response protein 88
NLRP3, Nacht, LRR: and PYD domains-containing protein 3
NO: Nitric oxide
NDRG1: N-myc downstream regulated gene 1
NF-HEV: Nuclear factor high endothelial venules
NFκB: Nuclear factor kappa-light-chain-enhancer of B cells
OPN: Osteopontin
PTX3: Pentraxin 3
PI3K: Phosphoinositide-3 kinase
PDK1: Phosphoinositide-dependent kinase-1
Akt: Protein kinase B
PTP: Protein tyrosine phosphatase
RA: Rheumatoid arthritis
RSPO3: R-spondin 3
SMAD1: SMAD family member 1
SMAD3: SMAD family member-3
SP1: Specificity protein 1
NFSA: Spontaneous fibrosarcoma
SSeCKs: Src-suppressed C-kinase substrate
SE: Status epilepticus
Th2: T-helper type-2
TF: Tissue factor
TOLLIP: Toll interacting protein
TIR8: Toll/IL-1R 8
TIR-domain: Toll/IL-1R domain
TLR: Toll-like receptor
TGF-β: Transforming growth factor β
TNF-α: Tumor necrosis factor alpha
u-PA: Urokinase-type plasminogen activator
VCAM-1: Vascular cell adhesion molecule-1
VEGF: Vascular endothelial growth factor
VEGFR2: Vascular endothelial growth factor receptor 2
VSMC: Vascular smooth muscle cells
vWF: von Willebrand factor
ZO-1: Zonula occludens-1.
